# The association between problematic cellular phone use and risky behaviors and low self-esteem among Taiwanese adolescents

**DOI:** 10.1186/1471-2458-10-217

**Published:** 2010-04-28

**Authors:** Yuan-Sheng Yang, Ju-Yu Yen, Chih-Hung Ko, Chung-Ping Cheng, Cheng-Fang Yen

**Affiliations:** 1 Department of Psychiatry, Kaohsiung Medical University Hospital, Tzyou 1st Rd., Kaohsiung 807, Taiwan; 2 Department of Psychiatry, Faculty of Medicine, College of Medicine, Kaohsiung Medical University, Chih-Chuan 1st Rd., Kaohsiung 807, Taiwan; 3 Department of Psychology, National Chung Cheng University, 168 University Road, Minhsiung Township, Chiayi County 62102, Taiwan; 4 Graduate Institute of Medicine, College of Medicine, Kaohsiung Medical University, Kaohsiung, Taiwan

## Abstract

**Background:**

Cellular phone use (CPU) is an important part of life for many adolescents. However, problematic CPU may complicate physiological and psychological problems. The aim of our study was to examine the associations between problematic CPU and a series of risky behaviors and low self-esteem in Taiwanese adolescents.

**Methods:**

A total of 11,111 adolescent students in Southern Taiwan were randomly selected into this study. We used the Problematic Cellular Phone Use Questionnaire to identify the adolescents with problematic CPU. Meanwhile, a series of risky behaviors and self-esteem were evaluated. Multilevel logistic regression analyses were employed to examine the associations between problematic CPU and risky behaviors and low self-esteem regarding gender and age.

**Results:**

The results indicated that positive associations were found between problematic CPU and aggression, insomnia, smoking cigarettes, suicidal tendencies, and low self-esteem in all groups with different sexes and ages. However, gender and age differences existed in the associations between problematic CPU and suspension from school, criminal records, tattooing, short nocturnal sleep duration, unprotected sex, illicit drugs use, drinking alcohol and chewing betel nuts.

**Conclusions:**

There were positive associations between problematic CPU and a series of risky behaviors and low self-esteem in Taiwanese adolescents. It is worthy for parents and mental health professionals to pay attention to adolescents' problematic CPU.

## Background

Cellular phone use (CPU) is an important part of life for many people. However, there have been increasing numbers of studies indicating that problematic CPU may complicate physiological and psychological problems [1-3]. In respective studies of physiological complications, the carcinogenic effects of CPU are still controversial [4,5], while the thermal [6] and non-thermal effects [7,8] of CPU have been confirmed. However, studies on the association between problematic CPU and psychological problems have been relatively few until now. Previously researchers have found that problematic CPU was associated with low self-esteem in adult populations [9] in addition to alcohol drinking [10] and depression [11] in adolescent populations. Because CPU is so prevalent in modern life, its association with psychological problems needs further study.

In general, adolescence is a critical transition for mental and physical development with vulnerability to ecological changes. Risk-taking had been proposed a common tendency in adolescence and some scholars regard it as one of the processes of normal development [12]. However, dangerous risk-taking that takes place when normal adolescent testing goes awry and an adolescent becomes locked into a pattern with largely negative consequences has been termed 'risky behavior' [13]. A series of risky behaviors may be noted in an adolescent during the period of adolescence and compromise the adolescent's development [14]. Furthermore, there was an explanatory psychosocial framework conceptualized by Jessor [14] which was based on the web of causations theory [15] to facilitate the understandings of adolescent risky behaviors. To our knowledge, there has been a lack of studies to survey the association between problematic CPU and risky behaviors in adolescents. If there are positive associations between problematic CPU and risky behaviors, it increases the possibility that problematic CPU may be one of risky behaviors in adolescents.

Self-esteem has been recognized as a powerful motivational force for adolescents. It is a sense of self-acceptance and respect for oneself, and it is based on a human need to be valued or to hold a positive self-evaluation [16]. Low self-esteem has been correlated with a wide range of negative psychological conditions [16]. Whether problematic CPU is associated with low self-esteem among adolescents needs further study.

We have developed the Problematic Cellular Phone Use Questionnaire (PCPU-Q) to evaluate adolescents' problematic CPU [11]. This study aimed to examine the associations between problematic CPU and a series of risky behaviors and low self-esteem in Taiwanese adolescents.

## Methods

The current investigation was based on data from the Project for the Health of Adolescents in Southern Taiwan, which has been described in detail elsewhere [11]. Based on the definitions of urban and rural districts in the Taiwan Demographic Fact Book [17] and school and grade characteristics, a stratified random sampling strategy was used with the final goal of ensuring that there was proportional representation of districts, schools and grades. Twelve junior high and 19 senior high/vocational schools were randomly selected from 120 junior high and 84 senior high/vocational schools in urban districts; likewise, 11 junior high and 10 senior high/vocational schools were randomly selected from 89 junior high and 56 senior high/vocational schools in rural districts. The classes of these schools were further stratified into three levels based on grades in both junior high and senior high/vocational schools. Then, 207 classes that contained a total of 12,210 adolescent students were randomly selected based on the ratio of students in each grade. We also recruited 76 adolescents (40 junior high school students and 36 senior high school students) and their parents into a pilot study to examine the 2-week test-retest reliability of all research instruments and the validity of problematic cell phone use and suicidal tendencies assessments.

### Assessment

The following seven questionnaires were given to each student in this study.

#### Problematic Cellular Phone Use Questionnaire (PCPU-Q)

We used the self-administered PCPU-Q to evaluate adolescents' problematic CPU. The 12-item PCPU-Q was developed according to the taxonomies of substance use dependence on the *DSM-IV-TR *[18] to determine the occurrence of symptoms of problematic CPU [11]. The first seven items inquired of participants whether they had or had not had the symptoms of problematic CPU in the preceding year, including: (1) Tolerance: a marked increase in the frequency and duration of CPU needed to achieve satisfaction, (2) Withdrawal symptoms without CPU, (3) CPU for a period of time longer or more frequent than intended, (4) Persistent desire and/or unsuccessful attempts to cut down or reduce CPU, (5) Excessive time spent on CPU or excessive effort spent on activities necessary to obtain cellular phone, (6) Giving up or reducing important social, academic, or recreational activities because of CPU, and (7) Continued heavy CPU despite knowledge of having a persistent or recurrent physical or psychological problem likely to have been caused or exacerbated by CPU. The last five items ascertained participants' subjective functional impairment in the preceding year caused by CPU. The 2-week test-retest reliability (Kappa) of the items on the PCPU-Q ranged from 0.410 to 0.778 (*p *< 0.001), and the Kappa between the participants' self-reports and the parents' reports ranged from 0.258 (*p *< 0.05) to 0.441 (*p *< 0.001) [11]. Cronbach's alpha of the seven items was 0.852 in this study. A previous study found that having four or more symptoms of problematic CPU on the first seven items of the PCPU-Q had the highest potential to differentiate those adolescents with functional impairment caused by CPU on the last five items of the PCPU-Q from those without functional impairment caused by CPU (Cohen Kappa = 0.463) [11]. Meanwhile, adolescents who had four or more symptoms of problematic CPU were more likely to have significant depression [11]. Thus, we used the first seven items of the PCPU-Q to represent the severity of problematic CPU, and adolescents who had four or more symptoms of problematic CPU were classified as having problematic CPU in this study.

#### Substance use

The four items of the Questionnaire for Experience in Substance Use (Q-ESU) was used to inquire the frequency of smoking, alcohol consumption, betel nut chewing, and illicit drug use during the preceding year [19]. Because most of participants had never used the substances evaluated in this study, the responses of participants were dichotomously divided to represent whether participants had smoked cigarettes, had drunk alcohol, and had chewed betel nuts every month during the preceding year, as well as whether they had ever used illicit drugs during the preceding year. The 2-week test-retest reliability of the items (Kappa) was 0.704-0.763 (*p *< 0.001).

#### Aggression

We used the following three questions from the Adolescent Aggressive Behaviors Questionnaire [20] to assess the occurrence of aggression during the preceding year: (1) "Have you ever hit or kicked someone on purpose?" (2) "Have you ever grabbed or shoved someone?" and (3) "Have you ever threatened to hurt someone or taken their things?" Another three questions were used to ask whether the participants had been the victims. The participants who answered "yes" to the first three questions and yes to the last three questions were classified as having perpetrated aggression to others and having been victimized by perpetrators, respectively. The 2-week test-retest reliability (Kappa) was 0.691 to 0.712 (*p *< 0.001) [21].

#### Suicidal tendencies

To assess the occurrence of suicide attempts and four forms of suicidal ideations during the preceding year, participants were invited to complete the questionnaire containing the following questions from the Epidemiological version of the Kiddie Schedule for Affective Disorders and Schizophrenia (Kiddie-SADS-E) [22]: (1) "Has there ever been a period of 2 weeks or longer when you thought a lot about death, including thoughts of your own death, somebody else's death, or death in general?" (2) "Has there ever been a period of 2 weeks or longer when you had a desire to die?" (3) "Have you ever thought of attempting suicide?" (4) "Have you had a plan for suicide?" and (5) "Have you ever attempted suicide?" Each question elicited a "yes" or "no" answer. The participants who answered "yes" to any of five questions were classified to having suicidal tendencies. The Kappa coefficient of agreement (Kappa) between participants' self-reported suicidal attempts and their parents' reports was 0.541 (*p *< 0.001) for Taiwanese adolescents [23].

#### Insomnia and short nocturnal sleep duration

We used an 8-item version of the Athens Insomnia Scale (AIS-8) to measure the participants' insomnia problems [24]. Each item of the AIS-8 was rated from 0 to 3, with 0 corresponding to "no problem at all" and 3 as a "very serious problem". The Cronbach's alpha in the present study was 0.669. In a pilot study, the 2-week test-retest reliability was 0.718 (p < 0.001) and the discriminating validity was good [25]. In this study, we classified the adolescents whose total AIS-8 score was higher than 10 (the 85th percentile of population) as having insomnia. The participants were also asked: "How many hours of sleep on average do you usually get every night during the recent month?" The 2-week test-retest reliability and correlation between the self-reported and parents' reported for the duration of nocturnal sleep (Pearson correlation's r) were 0.719 and 0.692 (p < 0.001), respectively [25]. We defined total nocturnal sleep duration below 6 hours (the 15th percentile of population) as short nocturnal sleep duration.

#### Low self-esteem

We used the 10-item Rosenberg Self-Esteem Scale (RSES) to assess current self-esteem [26]. A previous study found that the Cronbach's alpha was 0.861 and the 2-week test-retest reliability was 0.703 in Taiwanese adolescents [27]. We classified those with total RSES scores lower than 23 (the 15th percentile of population) as having low self-esteem.

#### Other questionnaires

We also collected participants' sex and age (<15 years old vs. ≧ 15 years old). The participants were requested to answer "yes" or "no" in self-reported questionnaires for experiences of tattooing, suspension from school, criminal record, and unprotected sexual behaviors.

### Procedure and statistical analysis

Research assistants explained the purpose and procedure of this study to the students in class, emphasizing respect for their privacy, and encouraged them to participate. Written informed assents were obtained from the adolescents beforehand. The protocol was approved by the Institutional Review Board of Kaohsiung Medical University. The IRB agreed that this study does not have to get the consent from the parents for their child's participation based on the three reasons. First, the Project for the Health of Adolescents is a routine survey of adolescent health conducted every three years and the adolescents could make the decision to complete the anonymous questionnaire by themselves. Second, this survey did no harm to the participants. Third, the IRB agreed that the results of this study will be beneficial to adolescents.

The adolescents were asked to anonymously complete the questionnaires based on the explanations of the research assistants and under their direction. All students received a gift that was worth 33 NT dollars (1 US dollar) at the end of the assessment. A total of 11,111 (91.0%) adolescents returned their written informed consent forms and completed the questions regarding problematic CPU without omission. Demographic characteristics, problematic CPU, risky behaviors and low self-esteem of the participants are shown in Table 1. Their mean age was 14.6 years (standard deviation: 1.7 years, range: 12-18 years).

**Table 1:** Demographic characteristics, problematic cellular phone use, and adverse mental health conditions.

Response item	Number (%)
Male (N = 11,111)	5,524 (49.7)
Age ≥ 15 years (N = 11,111)	5,431 (48.9)
Problematic cellular phone use (N = 11,111)	1,827 (16.4)
Perpetrated aggression (N = 11,111)	2,563 (23.1)
Victim of aggression (N = 10,990)	1,144 (10.3)
Had a tattoo (N = 10,975)	158 (1.4)
Insomnia (N = 10,922)	1,323 (12.1)
Short nocturnal sleep duration (N = 10,875)	1,388 (12.5)
Low self-esteem (N = 11,111)	1,771 (15.9)
Smoked cigarettes (N = 11,008)	632 (5.7)
Chewed betel nuts (N = 10,968)	142 (1.3)
Consumed alcohol (N = 10,993)	597 (5.4)
Used illicit drugs (N = 11,005)	151 (1.4)
Suspended from school (N = 11,046)	272 (2.4)
Had suicidal tendencies (N = 11,018)	3,245 (29.2)
Had a criminal record (N = 10,674)	335 (3.0)
Had unprotected sex (N = 10,398)	269 (2.4)

Descriptive data analysis was performed using the SPSS 14.0 statistical software. Because associations between problematic CPU and risky behaviors and low self-esteem may differ across gender and age, we divided our data set into four groups defined by gender and age. For each group, the associations were examined using univariate three-level logistic regression analyses using LISREL 8.8 (Scientific Software International Inc., Lincolnwood, IL, USA). Multilevel rather than ordinary logistic regression is conducted because students are considered as nested in classes and further nested in schools. In each logistic regression, risky behavior and self-esteem are predictors at student level but both their intercepts and slopes are treated as random effects in class and school level. A two-tailed *p *value associated with z test of fixed effects in three-level logistic regression was considered statistically significant if it is less than 0.05 (Tables 2 and 3). Meanwhile, we used the odds ratio (OR) and corresponding 95% confidence interval to estimate the effect size in the logistic regression analysis.

**Table 2:** The Relationships between problematic cellular phone use and adverse mental health conditions in girls and boys younger than 15 years

	Girls < 15 years		Boys < 15 years	
		
	z	OR (95% CI)	z	OR (95% CI)
Perpetrated aggression	5.481***	2.309 (1.711-3.114)	5.225***	2.282 (1.674-3.108)
Victim of aggression	5.299***	2.757 (1.895-4.015)	5.090***	2.083 (1.571-2.765)
Had a tattoo	2.509*	3.261 (1.059-10.034)	3.313**	5.099 (1.944-13.356)
Had insomnia	5.900***	2.440 (1.813-3.281)	4.716***	3.056 (1.921-4.860)
Short nocturnal sleep duration	3.794***	2.083 (1.426-3.047)	3.268**	2.217 (1.376-3.575)
Low self-esteem	6.993***	2.465 (1.914-3.174)	4.502***	2.368 (1.627-3.445)
Smoked cigarettes	6.415***	5.596 (3.307-9.469)	7.321***	4.380 (2.951-6.501)
Chewed betel nuts	0.403	4.527 (0.003-7058.586)	2.967**	3.736 (1.565-8.926)
Consumed alcohol	2.759**	2.522 (1.307-4.865)	2.378*	2.368 (1.163-4.816)
Used illicit drugs	3.225**	5.089 (1.893-13.681)	1.426	3.357 (0.636-17.708)
Suspended from school	-0.262	0.731 (0.070-7.668)	0.443	1.495 (0.252-8.855)
Had suicidal tendencies	4.646***	1.970 (1.480-2.620)	4.257***	2.517 (1.645-3.850)
Had a criminal record	0.821	1.448 (0.599-3.501)	2.513*	2.175 (1.186-3.987)
Had unprotected sex	5.412***	13.437 (5.244-34.432)	0.223	2.164 (0.002-1937.202)

*: *p *< 0.05; **: *p *< 0.01; ***: *p *< 0.001

**Table 3:** The Relationships between problematic cellular phone use and adverse mental health conditions in adolescent girls and boys at 15 years or older

	Girls ≥ 15 years		Boys ≥ 15 years	
		
	z	OR (95% CI)	z	OR (95% CI)
Perpetrated aggression	3.883***	1.870 (1.363-2.565)	4.663***	1.788 (1.401-2.282)
Victim of aggression	3.796***	2.026 (1.406-2.915)	2.801**	1.624 (1.157-2.280)
Had a tattoo	1.127	1.719 (0.670-4.411)	4.894***	5.349(2.732-10.465)
Had insomnia	4.845***	1.870 (1.452-2.408)	6.596***	2.509 (1.910-3.300)
Short nocturnal sleep duration	0.780	1.125 (0.836-1.516)	3.943***	1.933 (1.392-2.680)
Low self-esteem	4.078***	1.745 (1.335-2.280)	4.970***	1.984 (1.514-2.599)
Smoked cigarettes	2.202*	2.151 (1.088-4.255)	5.271***	2.467 (1.763-3.452)
Chewed betel nuts	0.049	1.129(0.009-137.827)	4.501***	3.414 (2.000-5.830)
Consumed alcohol	1.509	1.682 (0.856-3.304)	6.525***	2.904 (2.109-3.999)
Used illicit drugs	1.173	2.377 (0.559-10.125)	3.789***	3.593 (1.855-6.966)
Suspended from school	1.114	1.426 (0.763-2.664)	2.374*	2.188 (1.147-4.175)
Had suicidal tendencies	4.794***	1.835 (1.432-2.351)	3.931***	2.208 (1.487-3.277)
Had a criminal record	0.140	1.153 (0.158-8.423)	3.547***	2.586(1.530-4.371)
Had unprotected sex	4.715***	2.933 (1.876-4.586)	4.512***	3.149(1.914-5.186)

*: *p *< 0.05; **: *p *< 0.01; ***: *p *< 0.001

## Results

According to the definition described above, 1,827 adolescents (16.4%) had problematic CPU. Girls were more likely to have problematic CPU than boys (18.5% vs. 14.4%, χ^2 ^= 34.847, *p *< 0.001). Meanwhile, adolescents 15 years or older were more likely to have problematic CPU than adolescents younger than 15 years (19.9% vs. 13.2%, χ^2 ^= 90.667, *p *< 0.001). For the gender and age differences in those with problematic CPU, we examined the relationships of problematic CPU with risky behaviors and low self-esteem regarding gender and age, and the fixed effects of risky behaviors and self-esteem on CPU estimated by multi-level logistic analyses are shown in Table 2 and Table 3. The results indicated that in all girls and boys, those with problematic CPU were more likely to perpetrate aggression, be victims of aggression, have insomnia, have low self-esteem, smoke cigarettes, and have suicidal tendencies than those without problematic CPU. While girls younger than 15 years and all boys with problematic CPU were more likely to have tattoos, have short nocturnal sleep duration, and drink alcohol than those without problematic CPU, these significant relationships were not found in girls 15 years or older. While boys 15 years or older and all girls with problematic CPU were more likely to have unprotected sex than those without problematic CPU, we did not find such significant relationship in boys younger than 15 years. While boys with problematic CPU were more likely to have previous criminal records, and chew betel nuts than those without problematic CPU, those significant relationships were absent in girls. While girls younger than 15 years and boys 15 years or older with problematic CPU were more likely to use illicit drugs than those without problematic CPU, that relationship was not significant in girls 15 years or older and boys younger than 15 years. While boys 15 years or older with problematic CPU were more likely to have suspended from school than those without problematic CPU, no such significant relationship existed in other adolescents.

## Discussion

### Problematic CPU and involvement of aggression

In this study, we found a positive association between problematic CPU and aggression perpetration and victimization among all adolescent groups of different genders and ages. There are several possible explanations for the association. Firstly, those who have problematic CPU may have extended interpersonal contacts, which increase the risk to exposing themselves to physical conflicts with others. Secondly, researchers have found that there were positive associations between aggression involvement and several internalizing and externalizing problems [28]. Problematic CPU and aggression involvement, as well as other risky behaviors, might be the results of chaotic lifestyles. Thirdly, because most of the adolescents have no economical autonomy to buy their own cellular phone and to pay the monthly rental fee, the risk of involvement in interpersonal entanglement of money with subsequent violent aggression and victimization may increase.

### Problematic CPU and suicidal tendencies

This study found that problematic CPU was associated with suicidal tendencies among all groups of adolescents. Previous study results showed that adolescents with significant depression were more likely to have problematic CPU [11], which might partially account for the association between problematic CPU and suicidal tendencies. It is worthy to examine whether CPU has a devastating or protective effect on those with suicidal tendencies. While problematic CPU may worsen individuals' daily functioning and increase the risk of suicidal tendencies, those who used to use cellular phones may use cellular phones as a tool to call for help when they intend to hurt themselves.

### Problematic CPU and sleep problems

In this study problematic CPU was associated with insomnia in all groups of adolescents. Meanwhile, problematic CPU was associated with short nocturnal sleep duration in all groups of adolescents except for in girls 15 years or older. With a characteristic of being without predefined stopping points, intensive CPU may lead directly to a reduction in total sleep time by substituting for it. It may also be the contents of CPU that provokes sleep problems. Late and exciting CPU can cause high arousal and alertness in the brain, thus interfering with the calming effects that are necessary for sleep [29] and delaying the onset of sleep [30]. It is interesting that the significant association between problematic CPU and short nocturnal sleep duration was not found in girls 15 years or older in this study, which is not consistent with the results of a previous study [31]. Therefore, further surveys to address this are still needed.

### Problematic CPU and low self-esteem

In this study, we found a positive association between problematic CPU and low self-esteem among all adolescent groups, which was consistent with the results of a previous study [9]. Although the causal relationship between them can not be determined in this cross-sectional study, it is reasonable to suppose that the adolescents with negative self concepts may change their lives with CPU because of the immediate availability of emotional exchange and reassurance from CPU. Additionally, whether having a low self-esteem is the base to engage in risky behaviors in adolescents need further study.

### Problematic CPU and substance use

In this study, we found that problematic CPU was associated with smoking cigarettes in all adolescent groups, with drinking alcohol in girls younger than 15 years and all boys, with chewing betel nuts in boys, and with using illicit drugs in girls younger than 15 years and boys 15 years or older. Research also found that intensive CPU was associated with smoking cigarettes and excessive alcohol consumption [32]. Adolescent substance use has been widely considered as a risk factor for aggression [33,34]. Meanwhile, we also found a significant association between problematic CPU and involvement in aggression. Therefore, it seems reasonable to hypothesize that problematic CPU, substance use and aggression are an aggregation embedded in a chaotic lifestyle. Meanwhile, CPU could provide private and instant communication that is convenient to get illicit drugs for use. Further study is needed to survey the reasons for distribution of illicit drugs use in different adolescent groups regarding age and sex. While results of a previous study found that intensive CPU was considered to have a substitution effect on adolescent smoking [35], other studies did not support this result [10,36,37]. In addition, it is interesting to find that the association between problematic CPU and chewing betel nuts was not found in girls. The disfiguring effects may partially account for the results.

### Problematic CPU and unprotected sex

In this study, we found that problematic CPU was associated with unprotected sex in all adolescent groups except boys younger than 15 years. Although the causality could not be determined in this study, one possible explanation for the association was that the extension of interpersonal networks by CPU may increase the opportunities for close contact with potential sexual partners.

### Problematic CPU and tattooing, suspension from school and criminal record

In this study, we found that problematic CPU was associated with tattooing in all groups except for in girls 15 years or older. Meanwhile, problematic CPU was associated with previous criminal record in boys but not in girls. Additionally, problematic CPU was associated with suspension from school in boys 15 years or older. Tattooing is traditionally considered a form of deviant behavior in many Asian countries. The associations of problematic CPU with tattooing, suspension from school, and criminal records might further support that problematic CPU is a manifestation of disorganized lifestyles in adolescents. However, the association between problematic CPU and tattooing was not significant in the girls 15 years or older. One explanation is that girls 15 years or older may have greater concern about the disfiguring effects of tattooing than the other groups of adolescents. Meanwhile, gender and age may have moderating effects on the association between problematic CPU and suspension from school and previous criminal record.

Our study was the first survey to address the relationship of problematic CPU and series risky behaviors. The strength of this study is the large number of adolescents studied and the representative population-based origin of the study sample. However, some limitations of this study should be addressed. Firstly, although it is not a main aim of this study to determine the causal relationships between problematic CPU and risky behaviors, the cross-sectional research design of this study limited our ability to draw conclusions regarding the causal relationships. Secondly, because the data was provided by the adolescents, recall bias could not be completely excluded. Thirdly, this study recruited school population adolescent students as the research population; however, adolescents who had dropped out of school and were students in night schools who may have different patterns of risky behaviors were not recruited into this study. Future research may be focused on those not recruited in current study and on the CPU pattern and content in context with associated risky behaviors separately. Fourthly, there were small numbers of young girls having some kinds of risky behaviors. For example, only 24 (0.9%), 6 (0.2%), and 23 (0.8%) girls younger than 15 years had a tattoo, chewed betel nuts, and used illicit drugs, respectively. The small numbers of subjects with these risky behaviors limited the possibility to make the final conclusion about the relationships between CPU and these risky behaviors in young girls.

## Conclusions

The positive associations between problematic CPU and a series of risky behaviors and low self-esteem found in this study increase the possibility that problematic CPU is one of risky behaviors in adolescents. Because problematic CPU also may complicate the individuals' physiological and psychological problems [1-3], it is worthy for parents and mental health professionals to pay attention to adolescents' CPU.

## Competing interests

The authors declare that they have no competing interests.

## Authors' contributions

JY, CK and CY designed the study. CYen was responsible for supervising data collection. CK, CC and CY were responsible for data analysis. YY and CY drafted the manuscript. All authors critically revised the manuscript. All authors read and approved the final manuscript.

## Pre-publication history

The pre-publication history for this paper can be accessed here:

http://www.biomedcentral.com/1471-2458/10/217/prepub
